# Reproducibility of neutrophil percentages in pooled and non-pooled nasal lavage samples

**DOI:** 10.1186/1710-1492-7-S2-A26

**Published:** 2011-11-14

**Authors:** Dominik A Nowak, Penelope Ferrie, Paul K Keith

**Affiliations:** 1 University of Toronto Mississauga, Mississauga, Ontario, Canada, L5L 1C6; 2 McMaster University, Hamilton, Ontario, Canada, L8S 4L8

## Background

Neutrophils form a host’s main immediate immune response. COPD, ozone, infection and other factors have been shown to increase neutrophils found in blood, sputum and nasal lavage. Nasal lavage is used to collect cells and inflammatory mediators from the nasal cavity, and can be used quantify neutrophilic response to inhaled stimuli.

## Objective

To compare the reproducibility of neutrophil percentage in a single sample lavage, SSL, versus multiple sample lavage, MSL (3 lavages performed 15 minutes apart and pooled).

## Methods

Randomized crossover trial of nasal lavage performed on 4 visits 7-10 days apart, alternating between SSL and MSL done in 7 subjects with perennial allergic rhinitis, 7 with bilateral nasal polyposis and 7 controls.

## Results

The mean (±SEM) neutrophil percentage was not significantly different between the two methods (80 ±12 for SSL, 86 ±8 for MSL, p=0.2). At a minimum total cell count (TCC) cutoff of ≥20, the neutrophil percentage intraclass correlation (ICC) was similar but not sufficiently reproducible for SSL at 0.588 compared to MSL at 0.641. For samples with TCC≥100, the neutrophil percentage ICC of SSL was 0.93 (excellent correlation) and that of MSL was 0.676 (satisfactory). The evaluable samples for TCC≥100 were 60/84 for SSL and 67/84 for MSL. We previously demonstrated in the same experiment that a minimal TCC cutoff of ≥100 cells gave excellent eosinophil percentage ICC for both methods (>0.8).

## Conclusion

SSL ICC was superior to MSL in measuring nasal lavage neutrophil percentage. This method could be used to assess the effects of inhalant stimuli on the nasal cavity.

